# Efficient Transient Expression of Recombinant Proteins Using DNA Viral Vectors in Freshwater Microalgal Species

**DOI:** 10.3389/fpls.2021.650820

**Published:** 2021-04-07

**Authors:** Ashwini Malla, Sergio Rosales-Mendoza, Waranyoo Phoolcharoen, Sornkanok Vimolmangkang

**Affiliations:** ^1^Department of Pharmacognosy and Pharmaceutical Botany, Faculty of Pharmaceutical Sciences, Chulalongkorn University, Bangkok, Thailand; ^2^Research Unit for Plant-Produced Pharmaceuticals, Chulalongkorn University, Bangkok, Thailand; ^3^Laboratorio de Biofarmacéuticos Recombinantes, Facultad de Ciencias Químicas, Universidad Autónoma de San Luis Potosí, San Luis Potosí, Mexico; ^4^Sección de Biotecnología, Centro de Investigación en Ciencias de la Salud y Biomedicina, Universidad Autónoma de San Luis Potosí, San Luis Potosí, Mexico

**Keywords:** *Chlorella vulgaris*, *Chlamydomoans reinhardtii*, nuclear transformation, geminiviral vector, green algae, therapeutic proteins, quantification, western blot

## Abstract

The increase in the world population, the advent of new infections and health issues, and the scarcity of natural biological products have spotlighted the importance of recombinant protein technology and its large-scale production in a cost-effective manner. Microalgae have become a significant promising platform with the potential to meet the increasing demand for recombinant proteins and other biologicals. Microalgae are safe organisms that can grow rapidly and are easily cultivated with basic nutrient requirements. Although continuous efforts have led to considerable progress in the algae genetic engineering field, there are still many hurdles to overcome before these microorganisms emerge as a mature expression system. Hence, there is a need to develop efficient expression approaches to exploit microalgae for the production of recombinant proteins at convenient yields. This study aimed to test the ability of the DNA geminiviral vector with Rep-mediated replication to transiently express recombinant proteins in the freshwater microalgal species *Chlamydomonas reinhardtii* and *Chlorella vulgaris* using *Agrobacterium-*mediated transformation. The SARS-CoV-2 receptor binding domain (RBD) and basic fibroblast growth factor (bFGF) are representative antigen proteins and growth factor proteins, respectively, that were subcloned in a geminiviral vector and were used for nuclear transformation to transiently express these proteins in *C. reinhardtii* and *C. vulgaris*. The results showed that the geminiviral vector allowed the expression of both recombinant proteins in both algal species, with yields at 48 h posttransformation of up to 1.14 μg/g RBD and 1.61 ng/g FGF in *C. vulgaris* and 1.61 μg/g RBD and 1.025 ng/g FGF in *C. reinhardtii*. Thus, this study provides a proof of concept for the use of DNA viral vectors for the simple, rapid, and efficient production of recombinant proteins that repress the difficulties faced in the genetic transformation of these unicellular green microalgae. This concept opens an avenue to explore and optimize green microalgae as an ideal economically valuable platform for the production of therapeutic and industrially relevant recombinant proteins in shorter time periods with significant yields.

## Highlights

- A simple transformation method for *Chlorella* and *Chlamydomonas* is provided and feasible for scaling up recombinant protein production.
- The use of a geminiviral vector, which is normally used for transient plant transformation, has been successfully used for the expression of therapeutic proteins in algal systems.
- Microalgae are excellent, safe, and nutraceutically important organisms that are emerging as platforms for the development of various biological products.

## Introduction

Globally, there is a significant demand for the production of recombinant proteins due to their diversified properties in various applications, such as diagnosis, therapeutics, and industrial uses. Recombinant proteins are expressed in various hosts, such as bacteria (Swartz, 2001; Demain and Vaishnav, 2009; Ferrer-Miralles et al., 2009), yeast (Mattanovich et al., 2012; Kim et al., 2015), mammalian cells (Hacker and Balasubramanian, 2016; O’Flaherty et al., 2020), and plants (Mason and Arntzen, 1995; Cramer et al., 2000; Shanmugaraj et al., 2020a; Shanmugaraj and Phoolcharoen, 2021). Each expression system has its own advantages, disadvantages, and certain limitations, such as the availability of resources, inability to undergo molecular modifications, and requirement of sophisticated tools and techniques, resulting in toxic by-products and contamination risks (Barrera and Mayfield, 2013; Corchero et al., 2013). In the last decade, plants have emerged as an alternate platform for the expression of various functional recombinant proteins that have high therapeutic uses, with decreased manufacturing costs that are still in the pipeline for commercialization due to certain concerns related to environmental contamination, allergic reactions, and slow growth cycles (Salazar-González et al., 2015). Recombinant protein scale-ups in a safer way have drawn major attention to meet the current demand, with a focus on achieving higher quality and yields at mitigated production costs, thereby ensuring global coverage (Shanmugaraj and Phoolcharoen, 2021).

Microalgae comprise a diverse group of organisms with both prokaryotic and eukaryotic nature, making them an archetypal platform for recombinant technology. Microalgae are the source of a repertoire of natural biomolecules, with many of them having high protein, vitamin, and lipid contents, which have significant nutraceutical importance (Potvin and Zhang, 2010; Specht et al., 2010; Barrera and Mayfield, 2013; Yan et al., 2016; Bañuelos-Hernández et al., 2017; Ortega-Berlanga et al., 2018; Fayyaz et al., 2020; Sproles et al., 2021). Due to their ability to produce many compounds, algae have gained commercial and biotechnological interest, and some species are “generally regarded as safe” (GRAS) for their use as dietary supplements in humans and animals (Rosenberg et al., 2008). Microalgae represent the “best of both worlds” due to reasonable simple growth requirements with rapid growth and higher biomass and their ability to perform posttranslational modifications (Walker et al., 2005); thus, these are currently considered a promising platform for recombinant protein production with some particular advantages, including rapid transformation, no need for growth regulators, and the ability to properly synthesize complex proteins (Specht and Mayfield, 2014). Antigens from various pathogens, including the hepatitis B virus, *Plasmodium falciparum*, foot and mouth disease virus, *Staphylococcus aureus*, classical swine fever virus (CSFV), and influenza virus, have been produced in microalgae species, such as *Chlamydomonas reinhardtii* (freshwater), *Dunaliella salina* (marine), *Schizochytrium* spp. (marine), and *Phaeodactylum tricornutum* (Geng et al., 2003; Surzycki et al., 2009; Hempel et al., 2011; Gregory et al., 2012; Bayne et al., 2013). Photosynthetic unicellular algae, such as *C. reinhardtii* and *Chlorella vulgaris*, are most commonly used due to their high proteinaceous content with minimal nutrient requirements, such as salt-based media, carbon dioxide, and light, paving the way for much less difficult recombinant protein expression for many targeted applications (Potvin and Zhang, 2010; Tan et al., 2020). Microalgae are more advantageous for recombinant protein production, as they can be easily grown in contained bioreactors, minimizing the risk of airborne contaminants and protecting the environment from any potential flow of transgenes into the surroundings (Specht et al., 2010; Ahmad et al., 2020).

Successful transformation was achieved in microalgal species, such as *Chlamydomonas*, *Chlorella*, *Volvox*, *Haematococcus*, and *Dunaliella*, with very low levels of expression (Rosenberg et al., 2008). This may be due to barriers, such as thick cell walls, additional cellular membranes, target organelles, and the species being transformed (Barrera and Mayfield, 2013). Many transformation techniques, such as using cell wall-deficient strains (Hoffmann and Beck, 2005) and glass beads (Feng et al., 2008), electroporation (Feng et al., 2008), laboriously expensive mild cell disruption methods (Phong et al., 2018) such as bead milling (Günerken et al., 2015), ultrasonication (Piasecka et al., 2014), ionic liquids (Orr and Rehmann, 2016), microfluidization (Cha et al., 2011), pulsed arc technology (Goettel et al., 2013), cationic polymer-coated membranes (Yoo et al., 2014), and *Agrobacterium*-mediated transformation (Mayfield and Kindle, 1990; Kumar et al., 2004), have been developed for the stable nuclear expression of transgenes in a few microalgal species. Apart from these techniques, the transgene to be expressed requires codon optimization with construction in specific expression vectors using specialized promoters and selectable markers for higher expression levels in microalgae (Potvin and Zhang, 2010; Specht et al., 2010; Barrera and Mayfield, 2013). Although there has been a recent surge in the development of genetic engineering tools for microalgal transformation, one challenge for the field is to increase protein yields (Sproles et al., 2021). Hence, the present study aimed to develop an efficient transient expression approach for recombinant proteins using DNA viral vectors that target the nuclear genome in freshwater microalgal species.

The development and use of viral vectors have gained considerable attention in the recent decade for the heterologous expression of proteins in various hosts, most particularly in plant systems, and have emerged as a simple, easy, and effective mode of transformation with higher yields and good quality biological products (Chen et al., 2011; Dugdale et al., 2013; Salazar-González et al., 2015; Shanmugaraj et al., 2020a). One such system is based on geminiviral vectors that contain the replication elements Rep and Rep A from bean yellow dwarf virus (BeYDV), which are responsible for enhancing the expression levels of recombinant proteins. The vector backbone also has a cis-acting long intergenic region (LIR) and short intergenic region (SIR) elements with a duplicate enhancer and 35S CaMV constitutive promoter in the expression cassette. It has the 5′ untranslated region (UTR) from tobacco mosaic virus (TMV) and the P19 suppressor gene that is capable enough to target transgene expression in certain cases without the need for another vector. The use of geminiviral vectors has been proven efficient for transient expression (Chen et al., 2011) of various antigens, such as severe acute respiratory syndrome coronavirus 2 (SARS-CoV-2; Rattanapisit et al., 2020b); Ebola immune complex (Phoolcharoen et al., 2011); cholera toxin-B subunit; immunogen targeting the cytotoxic T-lymphocyte associated antigen-4 (Yiemchavee et al., 2021); growth factors, such as recombinant osteopontin (Rattanapisit et al., 2017, 2019), vascular endothelial growth factor (Bulaon et al., 2020), human epidermal growth factor (Hanittinan et al., 2020), and fibroblast growth factor (Rattanapisit et al., 2020a); and monoclonal antibodies (Shanmugaraj et al., 2020b), in *Nicotiana benthamiana*. Hence, these viral vectors, which have not been explored much in microalgae, can be used as an innovative approach in algal biotechnology to remove various barriers to nuclear transformation.

Here, the present study aimed to use the freshwater unicellular algal species *C. vulgaris* and *C. reinhardtii*, which are highly proteinaceous for genetically modifying their nuclear genome with viral vector transformation, using an antigen model SARS-CoV-2 receptor binding domain (SARS-CoV-2 RBD) and human basic fibroblast growth factor (bFGF) for biopharmaceutical expression. The SARS-CoV-2 RBD antigen and bFGF growth factor were particularly chosen because they have significant market demands and can be commercialized in the future by scaling up with this simple, rapid, and efficient nuclear transformation technique. We explored the potential of the geminiviral expression vector pBYR2eK2Md (pBYR2e) for nuclear transformation in microalgal species for recombinant protein production. In this context, both SARS-CoV-2 and bFGF were efficiently expressed by using the geminiviral vector in *C. vulgaris* and *C. reinhardtii*. The expression of recombinant SARS-CoV-2 and bFGF was determined by western blotting and quantified by enzyme-linked immunosorbent assay (ELISA) in both species. This study provides a proof of principle for the rapid production of biological compounds in microalgal species using DNA viral vectors that address the safety, cost, and easy scale-up in comparison with other production platforms and genetic engineering methods.

## Materials and Methods

### Microalgae Growth Conditions

An axenic culture of *C. reinhardtii* was kindly provided by Professor Sergio Rosales Mendoza, UASLP, Mexico. *C. vulgaris* was obtained from the Thailand Institute of Scientific and Technological Research (TISTR), Pathum Thani, Thailand. *C. reinhardtii* and *C. vulgaris* were grown in Tris acetate phosphate (TAP) and f/2 media without silicates, respectively, from PhytoTech Labs. The two axenic microalgal cultures were incubated at 28 ± 1°C with cool fluorescent lamps at a photosynthetic photon flux density of 80–100 μmol/m^2^/s and a 16-h:8-h light:dark cycle on a rotary shaker (150 rpm) for 1 week. Every week thereafter, a subculture was started by routine transfer into fresh culture medium. For transformation, the *C. reinhardtii* and *C. vulgaris* species were inoculated at 5% (v/v) in fresh TAP and f/2 media and cultivated for 4–6 days at 150 rpm until growth approached the log phase (OD at 680 nm of 0.6–0.8).

### Vector Construction and Transformation Into *Agrobacterium tumefaciens*

The RBD region in the spike protein of SARS-CoV-2 (GenBank accession number: YP_009724390.1; F318-C617) was taken from NCBI and commercially synthesized (Genewiz, Suzhou, China). The bFGF nucleotide sequence (accession no: AAQ73204.1) was retrieved from GenBank and synthesized by GeneArt Gene Synthesis (Thermo Fisher Scientific). Furthermore, RBD and bFGF were fused with an 8X His tag at the C-terminus and cloned into a geminiviral vector (pBYR2e) by using *Xba*I and *Sac*I restriction enzymes to create pBYR2e:SARS-CoV-2 RBD and pBYR2e:FGF, respectively. The expression vectors were transformed into *Agrobacterium tumefaciens* strain GV3101 by electroporation, and the resulting strains were confirmed by PCR (Rattanapisit et al., 2020a,b). *Agrobacterium* harboring the RBD and FGF genes was used for transient transformation in microalgal species.

### Transformation of Microalgae With DNA Viral Vectors Harboring SARS-CoV-2 RBD and bFGF

The *C. reinhardtii* and *C. vulgaris* cultures were grown as per the conditions mentioned in section “Microalgae Growth Conditions.” Nuclear transformation was carried out in both *C. reinhardtii* and *C. vulgaris* species using 1.0 ml of *A. tumefaciens* culture harboring the SARS-CoV-2 RBD gene or bFGF gene with OD_600 *nm*_: 1.0 for 100 ml of each algal culture with OD_680 *nm*_: 0.6–0.8. Each culture medium was supplemented with 100 μM acetosyringone for efficient T-DNA transfer. Twenty-four hours postinfection, cefotaxime was added at a final concentration of 250 mg/l. The expression of the SARS-CoV-2 RBD and FGF in the transiently transformed microalgae was analyzed by collecting biomass samples at 24, 48, and 72 h postantibiotic addition. The cells were harvested by centrifugation at 5,000 rpm for 10 min at 4°C. The supernatant was discarded, and the pelleted biomass was used for protein extraction.

### Protein Extraction and Analysis

Recombinant protein expression was analyzed by western blotting. Cell samples collected at different time intervals were used for protein extraction. One hundred milligrams of fresh biomass was homogenized in 200 μl of extraction buffer containing 750 mM Tris–HCl at pH 8.0 with 15% sucrose, 100 mM β-mercaptoethanol, and 1 mM PMSF as described previously (Bañuelos-Hernández et al., 2017). The extracts were sonicated for four pulses for 10 s with an interval of 5 s. Samples were subsequently centrifuged at 8,000 rpm for 15 min, and the supernatants were collected for further analysis. Total soluble proteins (TSPs) in the extracts were determined by the Bradford method. TSP (15–20 μg, approximately 50 μl) was mixed with 4X reducing loading buffer, denatured by boiling for 10 min at 95°C, and subsequently subjected to sodium dodecyl sulfate-polyacrylamide gel electrophoresis (SDS-PAGE) analysis. Gels were blotted onto 0.45-μm PVDF nitrocellulose membranes. Protein transfer was performed using a Bio-Rad electroblotter for 1 h at 100 V in a methanol-based transfer buffer. The nitrocellulose blot was further processed for immune analysis. After blocking with 5% blocker (Bio-Rad) dissolved in 1X PBS for 1 h, the blots were incubated at 4°C overnight with a rabbit anti-His antibody tagged with HRP conjugate (ab1187, Abcam, United Kingdom) at a dilution of 1:5,000 in 3% fat-free milk dissolved in 1X PBS. The blot was washed three times with PBST (PBS containing 0.05% Tween 20). Antigen detection was revealed by incubating the blots with SuperSignal West Pico Plus Chemiluminescent Substrate solution following the instructions from the manufacturer (Thermo Fisher Scientific, http://www.thermoscientific.com) and exposing the film in the dark.

### Quantification by ELISA

Severe acute respiratory syndrome coronavirus 2 RBD antigen (250 μg/ml) purified from recombinant *N. benthamiana* was used as the positive control. For quantitative ELISA, crude protein extracts from *C. reinhardtii* and *C. vulgaris* were diluted in 0.2 M carbonate buffer (pH 9.6). The diluted sample was added to an ELISA plate for protein adsorption overnight at 4°C. After blocking with 5% fat-free dry milk solution for 2 h at room temperature, the plates were incubated with anti-RBD serum (1:3,000) collected from sheep immunized with synthetic partial RBD peptide (CLKPFERDISTEIYQAGSTPCNGVEGFNCYFPLQ) overnight at 4°C. The plates were subsequently incubated with horseradish peroxidase-conjugated anti-sheep IgG at a dilution of 1:5,000 (A3415, Sigma-Aldrich) for 2 h at room temperature. A colorimetric reaction was induced by the addition of the TMB substrate solution (Promega, United States) with 30 min of incubation at 25°C. The absorbance values were measured at 450 nm. Standard curves were constructed using pure RBD protein from *N. benthamiana* to estimate the expression levels.

The bFGF expressed in transformed *C. vulgaris* and *C. reinhardtii* was quantified using the human FGF basic ELISA Kit (R&D Systems). The protocol was followed according to the manufacturer’s instructions.

## Results

### Expression of Recombinant SARS-CoV-2 RBD and bFGF in Microalgal Species

**FIGURE 1:**
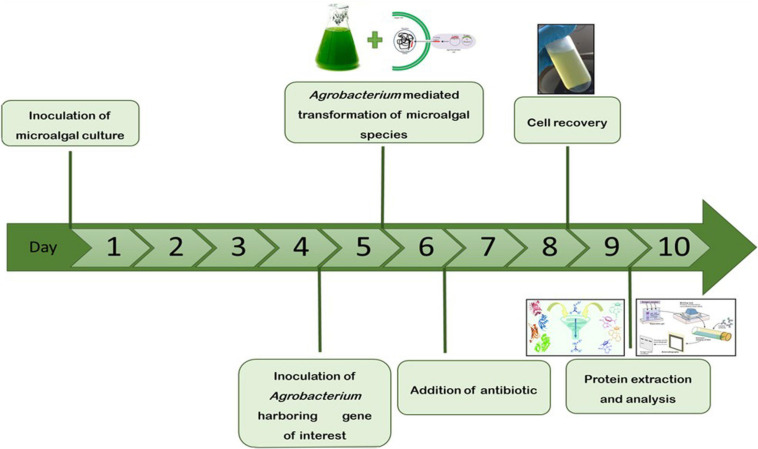
Schematic representation and timeline for the production of recombinant proteins in microalgae by transient gene expression.

**FIGURE 2:**
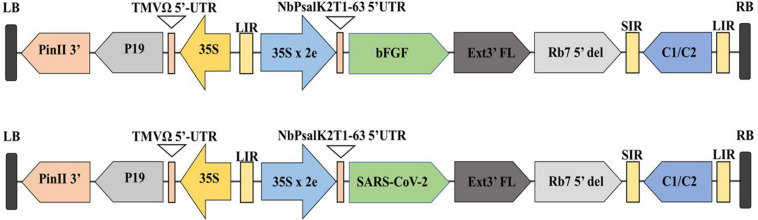
Diagrammatic representation of the T-DNA regions of the expression vector pBYR2eK2Md (pBYR2e). Modified from (Rattanapisit et al., 2020a,b). The bFGF and RBD genes were cloned into geminiviral vector using Xbal and Sad sites. RB and LB: The right and left borders of the T-DNA region in Agrobacterium; P35S: Cauliflower Mosaic Virus (CaMV) 35S promoter; P19: the RNA silencing suppressor from tomato bushy stunt virus (TBSV); NbPsalK2Tl-63 5′UTR: 5′ untranslated region; RBD: SARS-CoV-2 RBD; FGF: basic fibroblast growth factor gene; Ext3’FL: 3’ region of tobacco extension gene; Rb7 5′: 5′ of the Rb7 matrix attachment region/scaffold attachment region; SIR: short intergenic region of BeYDV: LIR: long intergenic region of BeYDV; C2/C1: Bean Yellow Dwarf Virus (BeYDV) ORFs Cl and C2 encoding for replication initiation protein (Rep) and Rep A: TMVfi 5′-UTR: 5′ untranslated region of tobacco mosaic virus Ω; Pinll 3’: the terminator from potato proteinase inhibitor II gene.

The microalgae *C. vulgaris* and *C. reinhardtii* were grown in f/2 and TAP media, respectively, by adding 5% inoculum. The cultures were allowed to grow until the absorbance at 680 nm reached 0.8 and then transformed with *Agrobacterium* harboring SARS-CoV-2 RBD or bFGF in both microalgal species. The sequential flow representation and timeline for the production of recombinant proteins in microalgae are depicted in Figure 1. The geminiviral vector pBYR2e used for the current study drives the expression of transgenes under the control of the 35S promoter along with the suppressor P19 RNA silencing gene for higher protein expression (Diamos and Mason, 2019). The structural orientation of the vector pBYR2e used in this study is represented in Figure 2. *Agrobacterium-*mediated transformed cultures of *C. vulgaris* and *C. reinhardtii* with SARS-CoV-2 RBD and bFGF were collected at three time points (24, 48, and 72 h after antibiotic addition). The algal cultures were recovered by centrifugation, and the fresh biomass was weighed. The total soluble protein concentration was estimated by the Bradford assay using bovine serum albumin (Sigma) as the standard.

### Sodium Dodecyl Sulfate-Polyacrylamide Gel Electrophoresis and Western Blot Analysis

**FIGURE 3:**
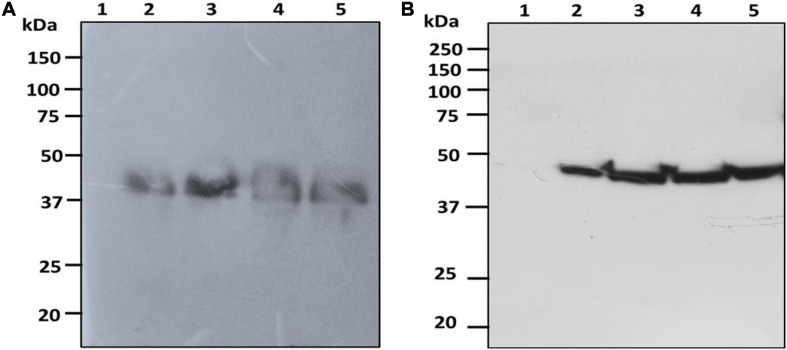
Western blot analysis of RBD protein of SARS-CoV-2 produced in microalgal species. The crude proteins were extracted from *Chlorella* **(A)** and *Chlamydomonas* **(B)** and the expression of RBD protein was analyzed on SDS-PAGE followed by Western blot probed with a rabbit anti-his antibody conjugated with HRP. Lane 1: Wild Type; Lane 2: Sample collected after 24 h of Agrobacterium transformation and cefotaxime addition; Lane 3: Sample collected after 48 h; Lane 4: Sample collected after 72 h; Lane 5: Positive control (Agro-infiltrated crude extract of *N. benthamiana*).

The fresh biomass collected from transformed cultures of *C. vulgaris* and *C. reinhardtii* with SARS-CoV-2 and bFGF was lysed and used for protein extraction. The expression of the recombinant RBD and FGF proteins was evaluated by SDS-PAGE and western blot analysis. The RBD protein was observed at the expected molecular weight of approximately 38 kDa in the western blot for both *C. vulgaris* and *C. reinhardtii* (Figure 3; lanes 2–4). The 3-day postinfiltrated leaf extract of *N. benthamiana* was used as the positive control (Figure 3; lane 5), and the wild-type extracts of both algae species did not show any protein expression (Figure 3; lane 1). RBD protein expression was observed in all the samples collected at 24, 48, and 72 h in both freshwater microalgal species and was further confirmed by immunoblotting assay.

**FIGURE 4:**
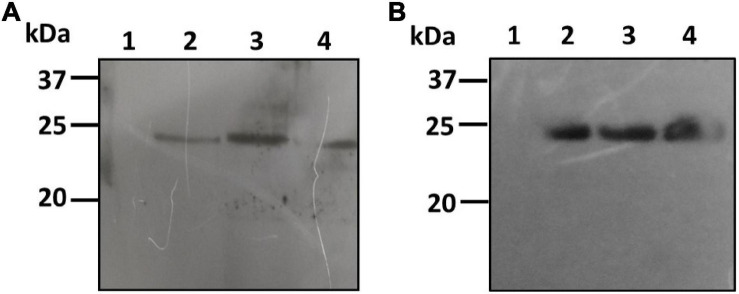
Western blot analysis of hFGF protein expressed microalgal species. The crude proteins were extracted from *Chlorella* **(A)** and *Chlamydomonas* **(B)** and the expression of hFGF protein was analyzed on SDS-PAGE followed by Western blot probed with a rabbit anti-his antibody conjugated with HRP. Lane 1: Wild Type; Lane 2: Sample collected after 24 h of *Agrobacterium* transformation and cefotaxime addition; Lane 3: Sample collected after 48 h; Lane 4: Sample collected after 72 h.

The pBYR2e:bFGF-transformed culture extracts of both *C. vulgaris* and *C. reinhardtii* were also analyzed by PAGE and western blotting using the rabbit anti-His antibody as an HRP conjugate for recombinant protein expression. The bFGF protein was observed at a molecular weight of approximately 24 kDa by western blotting for both *C. vulgaris* and *C. reinhardtii* (Figure 4; lanes 2–4). The extracts from both of the wild-type test algae did not show any protein expression (Figure 4; lane 1). FGF protein expression was low and was observed in all the samples collected at 24, 48, and 72 h in both microalgal species and was further quantified by ELISA. These results confirmed that geminiviral vectors can be used for the genetic transformation of green microalgal species. The expressed recombinant proteins were stable for a period of 1 month, and an in-depth molecular analysis needs to be performed to establish their strength, efficiency, and safety.

### Quantification of RBD and bFGF Produced in Freshwater Microalgal Species

**FIGURE 5:**
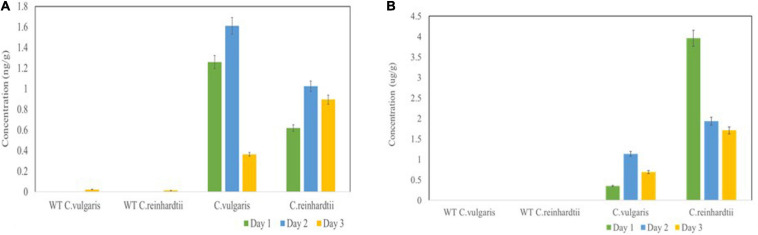
Expression levels of bFGF **(A)** and SARS-CoV-2 **(B)** in microalgal species was measured by ELISA. Microalgal species were transformed with *Agrobacterium* harboring the expression vector and the samples were collected on Day 1, 2, 3 and the expression was analyzed. The result was presented on the fresh weight (FW) basis. The data are expressed as mean ± standard deviation of two biological replicates with each sample taken in duplicates.

Basic fibroblast growth factor yields were determined by capture ELISA using a commercial kit. The bFGF protein yields at 24, 48, and 72 h reached 1.26, 1.61, and 0.365 ng/g fresh weight (FW) in *C. vulgaris* and 0.62, 1.025, and 0.895 ng/g FW in *C. reinhardtii*, respectively (Figure 5A). Although FGF expression was low, it accounted for 0.02–1% of TSP in both tested green algae, thereby confirming the authenticity and proper molecular channeling of the expressed protein.

We also further quantified the recombinant RBD expressed in both tested algae species by ELISA using the purified RBD (125 μg/ml) from *N. benthamiana* as a standard. The RBD standard along with crude extracts from transformed *C. vulgaris* and *C. reinhardtii* was coated on 96-well plates and further incubated with anti-RBD serum from sheep immunized with a peptide derived from the RBD. The algae-made RBD showed specific binding with the anti-RBD antibody, whereas negative controls did not show any binding. These results confirmed that the use of DNA viral vectors can be used to produce recombinant antigens in green algae. The transformed *C. vulgaris* accumulated the RBD protein at concentrations of 0.35, 1.14, and 0.69 μg/g FW at 24, 48, and 72 h, respectively (Figure 5B), showing a high level of expression on day 2. *C. reinhardtii* showed a higher expression of RBD at concentrations of 3.96, 1.94, and 1.71 μg/g FW on days 1, 2, and 3, respectively (Figure 5B). Overall, the recombinant RBD protein expressed in the tested algae species ranged from 0.43 to 3.6% TSP. For the quantification studies, two biological replicates were performed, and each sample was subjected to two technical replicates. The data are presented as the means ± standard deviation.

## Discussion

The demand and use of recombinant proteins have become powerful tools globally due to their wide significance and important application in industry, diagnosis, and therapeutics (Rosales-Mendoza et al., 2020a). Hence, there is an immense need for the development and production of recombinant proteins in large quantities using various eukaryotic expression systems to provide several solutions, such as cost reduction and bioavailability of these biological substances in low- and middle-income countries in a safer and efficient way, by expanding the potential biosystems (Rosales-Mendoza et al., 2020b). Biopharmaceuticals are mainly produced in expression systems that include mammalian cells, bacteria, insect cells, yeast, and plants that are capable of meeting market demands with sufficient production levels but with some limitations (Shih and Doran, 2009; Dhara et al., 2018; Kis et al., 2019). The most commonly used expression system involves Chinese hamster ovary cells and plants for the production of various complex biomolecules that include vaccines, antibodies, hormones, clotting factors, enzymes, growth factors, and cytokines. The mammalian system requires complex nutritional requirements that involve high production costs with a high chance of contamination, requiring stringent sterility maintenance, whereas plant systems are not expensive, but there are certain technical and regulatory concerns for commercial scale production that remain to be addressed (Chen and Davis, 2016; Dhara et al., 2018; Burnett and Burnett, 2020; Shanmugaraj et al., 2020a).

Microalgae are diverse unicellular eukaryotic organisms with interesting properties that make them attractive hosts for recombinant protein production (Specht et al., 2010; Specht and Mayfield, 2014); these organisms are a rich source of high-value compounds, such as proteins, lipids, polyunsaturated fatty acids, and carotenoids, involved in the light-driven synthesis of therapeutic compounds at lower operating costs for upstream and downstream processes (Ahmad et al., 2020; Rosales-Mendoza et al., 2020b). Photosynthetic microalgae, such as *C. reinhardtii* and *C. vulgaris*, are considered safe for consumption as dietary supplements and form an attractive platform for the synthesis of high-value heterologous proteins due to their many beneficial attributes, such as ease of cultivation, lack of pathogens, and simple media components, allowing them to easily grow in containment photobioreactors under sterile conditions (Rosenberg et al., 2008; Potvin and Zhang, 2010; Specht et al., 2010; Yaakob et al., 2014; Rasala and Mayfield, 2015; Salazar-González et al., 2015). However, recombinant protein production levels are frequently very low, and thus, refinement of expression technologies is needed to enhance yields.

Hence, the present study was focused on the use of viral vectors for transient nuclear expression of recombinant proteins, which has not been explored previously in *C. reinhardtii* and *C. vulgaris*. A geminiviral expression system was selected based on replication elements from the BeYDV and the CaMV 35S promoter and enhancer (Chen et al., 2011; Diamos and Mason, 2019). The pBYR2e vector carrying the SARS-CoV-2 and bFGF proteins in this study was successfully transformed in *N*. *benthamiana*, a tobacco species with yields of 8 and 2.16 μg/g FW, respectively, 3 days postinfiltration (Rattanapisit et al., 2020a,b). The expression levels of recombinant proteins produced are high in plants, as the vector is particularly designed for plant systems, but the regulations, environmental concerns, and safety approval for oral consumption still need to be resolved (Streatfield, 2005; Laere et al., 2016).

The microalgal species *C. reinhardtii* and *C. vulgaris* were transiently transformed with *Agrobacterium* strains carrying the geminiviral vector called pBYR2e coding for the proteins of interest. To assess the potential of the expression system based on DNA replicons, we selected two biopharmaceuticals: SARS-CoV-2 RBD, which is an antigen derived from the spike protein of the novel coronavirus, and bFGF, which is a growth factor. These proteins were successfully expressed in both photosynthetic microalgal species, thereby proving the proof of principle to use these DNA viral vectors as a functional and efficient approach for transient expression of proteins without the involvement of any laborious processes. In a similar study by Bañuelos-Hernández et al. (2017), the Algevir system for transient expression of GP1 from the Zaire Ebola virus and LTB from *Escherichia coli* were assessed in *Schizochytrium* species, revealing the robustness of using viral vectors that employ viral replication elements for microalgal transformation (Bañuelos-Hernández et al., 2017). The maximum expression levels were attained for the SARS-CoV-2 RBD after 48 h of transformation, reaching maximum yields of 1.14 and 1.94 μg/g FW in *C. vulgaris* and *C. reinhardtii*, respectively. FGF expression was also found to be highest after 2 days of transformation with accumulation of 1.61 and 1.025 ng/g FW in *C. vulgaris* and *C. reinhardtii*, respectively. The *Schizochytrium* species that was transformed with the Algevir vector system showed maximum expression levels after 48 h postinduction with accumulation of 1.25 mg/g FW for GP1 and 0.12 mg/g FW for LTB (Bañuelos-Hernández et al., 2017). The recombinant human CL4mAb against the hepatitis B surface antigen (HBsAg) fused to green fluorescent protein and a tetrapeptide was expressed in the *P. tricornutum* nuclear genome, accumulating up to 8.7% TSP (Hempel et al., 2011). In the *C. reinhardtii* nuclear genome, the human erythropoietin (EPO) protein was expressed with the hsp70A/rbcS2 chimeric promoter (Eichler-Stahlberg et al., 2009). The expression of HIV-1 protein P24, which is a vaccine candidate for AIDS, was successfully achieved in *Chlamydomonas* using the PSAD promoter with 0.25% of the total cellular protein (Barahimipour et al., 2016). *P. tricornutum* was also successful in expressing recombinant mAb against a nucleoprotein part of the potent Marburg virus (Hempel et al., 2017). However, the expression of these recombinant proteins in algae is not comparable with those produced in tobacco plant systems. However, our study is the first report that has proven that the same plant vector can be used in microalgae transformation and that host-specific codon optimization and other genome editing techniques (Sproles et al., 2021) could enhance the yield of expression levels, which need to be further investigated.

One strength of using green algae is the possibility of implementing the delivery of therapeutic proteins orally. Algal cells are known to be natural green factories that can emerge as excellent organisms with highly valuable and significant products through gene transformations (Georgianna and Mayfield, 2012; Dyo and Purton, 2018). These eukaryotic microorganisms, which are regarded as safe, unlike the bacterial system, are capable of undergoing complex protein folding and modifications to form active proteins that are specifically important for human use as antigens and antibodies (Ma et al., 2020). Each system needs to be evaluated for its attributes before developing an oral vaccine delivery platform (Rosales-Mendoza et al., 2016). From this study, we predict the use of DNA viral vectors to transiently transform essential edible microalgae for the expression of various therapeutically important proteins and use them as oral delivery vehicles. It is only a preliminary yet significant concept, and furthermore, detailed studies are needed to test its efficacy, safety, and limitations.

Although nuclear and chloroplast transformation methods are available for microalgal species, these technologies have been frequently associated with low yields and genetic instability of transformed clones associated with gene silencing and genetic rearrangements and require lengthy processes for the transformation and selection of candidate clones. Advances in technologies that target chloroplast transformation have led to improvements in protein yields (Mayfield and Kindle, 1990; Surzycki et al., 2009; Specht and Mayfield, 2014). Chloroplast-based expression offers high protein productivity due to the high expression derived from the high copy number of transformed genomes per chloroplast and the general absence of gene silencing with no position effects; the integration is site-directed and mediated by homologous recombination (Bock and Warzecha, 2010). A limitation to consider for chloroplast-expressed biopharmaceuticals is the lack of complex posttranslational modifications, such as glycosylation, that are relevant for some biopharmaceuticals. In addition, it should be considered that protein secretion into culture media is not possible under chloroplast-based expression. Currently, yields in chloroplast expression are typically in the 0.02–5% TSP range with notable exceptions (42% of TSP of VP28) (Surzycki et al., 2009). Few other algal species have shown yields up to 3 mg/l of bovine milk amyloid A protein by chloroplast expression and 15 mg/l of recombinant protein secretion in culture media by nuclear expression (Lauersen et al., 2013; Gimpel et al., 2015; Ramos-Vega et al., 2018). Recombinant hemagglutinin proteins from the influenza virus have been expressed in the microalga *Schizochytrium* sp. in a secretion modality with yields up to 20 mg/l (Bayne et al., 2013).

In comparison, the use of viral vectors for transient expression in microalgae is considered more attractive since its features could overcome some of the limitations of the stable nuclear and chloroplast approaches. Nuclear transformation allows the cellular machinery to undergo molecular modifications, whereas the intracellular accumulation of antigens allows the whole cell to serve as a vaccine delivery vehicle along with the host’s endogenous compounds (Bañuelos-Hernández et al., 2017). In addition, the present study involves the use of the geminiviral vector pBYR2e, which offers enhanced expression of target proteins in the presence of duplicate enhancers and offers a short production time period.

In summary, the use of DNA viral vectors for genetic engineering in algal species for transient expression of therapeutic proteins is a versatile method that has many advantages, such as a short production time with significant protein yields without the involvement of cumbersome procedures, such as stable nuclear and chloroplast transformation techniques. Another advantage is that there will be no genetic instability of the stable transformants, as is the case of nuclear and chloroplast stable transformation of microalgae (Bañuelos-Hernández et al., 2017). Therefore, the DNA viral vector system was initially tested with the photosynthetic unicellular algal species *C. vulgaris* and *C. reinhardtii*, which can be easily grown on a large scale and employ transient transformation (Qu et al., 2013; Song et al., 2015). In conclusion, this study provides a proof of concept for the use of DNA viral vectors with replication elements as a robust approach for the simple and efficient transient expression of recombinant proteins in microalgae, which will open new avenues in the genetic engineering of other microalgal species. The expression levels of SARS-CoV-2 RBD and bFGF in both green algal species were significant, paving the way for scale-up and detailed studies on potency, strength, and safety in a cost-effective manner to reach market targets. Furthermore, these edible green *Chlamydomonas* and *Chlorella* species can be employed for the production of other valuable metabolites using geminiviral vector transformation with advanced genome editing methods to enhance the quality and yields of the bioproducts, thus economically and environmentally broadening the feasibility of the algal platform.

## Data Availability Statement

The original contributions presented in the study are included in the article/supplementary material, further inquiries can be directed to the corresponding author/s.

## Author Contributions

SV conceived and designed the experiments and revised the manuscript. SV, SR-M, and WP supervised the study. AM carried out the transformation in microalgal species and performed the protein expression and quantification by ELISA, analyzed the data, and wrote the manuscript. All authors read and approved the final version of the manuscript.

## Conflict of Interest

The authors declare that the research was conducted in the absence of any commercial or financial relationships that could be construed as a potential conflict of interest.
